# Lived experiences of first-time mothers receiving postpartum social support at Banadir Hospital, Mogadishu, Somalia: a phenomenological qualitative study

**DOI:** 10.1186/s12884-026-08875-y

**Published:** 2026-02-26

**Authors:** Abdullahi Abdiasis Ibrahim, Florence Githinji, Regina Ndagire, Angela Namwanje, Aaron Ssenyondo, Agnes Agwang, Chloe Nampiima, Fiona Atim

**Affiliations:** 1 School of Public Health, Clarke International University, P.O. BOX 7782, Kampala, Uganda; 2 School of Business and Applied Technology, Clarke International University, P.O. BOX 7782, Kampala, Uganda; 3 School of Nursing, Clarke International University, P.O. BOX 7782, Kampala, Uganda; 4 Quality Assurance Department, Clarke International University, P.O. BOX 7782, Kampala, Uganda; 5 School of Postgraduate Studies Research and Innovations, Clarke International University, P.O. BOX 7782, Kampala, Uganda; 6 University of Bosaso, Puntland, Somalia

**Keywords:** Postpartum care, Social support, First-time mothers, Banadir Hospital, Somalia

## Abstract

**Background:**

The postpartum period is crucial for maternal and newborn health but remains a high-risk phase in low-resource settings like Somalia, where psychosocial support is often lacking. Social support (including practical help), emotional care, and health education are essential for recovery and well-being. Despite Somalia’s high maternal mortality, little research has focused on first-time mothers’ experiences. This study explored their experiences of postpartum support at Banadir Hospital in Mogadishu.

**Methods:**

An exploratory phenomenological design guided this study. Data was collected through 16 individual interviews, two focus group discussions (12 participants), and 10 key informant interviews with healthcare workers, totaling 38 participants. Purposive sampling ensured the selection of participants with relevant experiences, and data saturation determined the final sample size. Interviews were conducted in Somali, audio-recorded with consent, and transcribed verbatim. Inductive thematic content analysis was performed using NVivo 11. Methodological rigor was maintained through triangulation, member checking, reflexive journaling, peer debriefing, and a comprehensive audit trail.

**Results:**

Three major themes emerged that reflected the postpartum social support experiences of first-time mothers. They received initial physical support primarily from family members, including mothers, mothers-in-law, and spouses. However, this assistance was limited and diminished significantly after the first month due to financial constraints and competing responsibilities. Emotional support was insufficient and primarily provided by healthcare workers rather than family, reflecting limited awareness of postpartum mental health needs and signs of depression. Informational support was critically inadequate, with mothers reporting unmet needs for structured guidance on newborn care, infant danger signs, and maternal health; they relied predominantly on informal sources, including family and neighbors, who often provided conflicting or inaccurate information. Overall, although postpartum support mechanisms exist, they are fragmented, inconsistent, and insufficient to meet first-time mothers’ holistic physical, emotional, and informational needs.

**Conclusion:**

The study highlights the urgent need for policy action to enhance postpartum care systems. This includes workforce capacity building and structured follow-up in fragile settings like Somalia. Strengthening postpartum social support through trained health workers, family engagement, and peer programs can improve maternal and neonatal outcomes.

**Supplementary Information:**

The online version contains supplementary material available at 10.1186/s12884-026-08875-y.

## Introduction

The postpartum period, defined as the time immediately following birth to six weeks (42 days) thereafter, represents a critical window for both maternal and neonatal health [1]. During this interval, mothers undergo significant physiological changes, including uterine involution, hemodynamic stabilization, and hormonal rebalancing, while infants complete the vital developmental transitions required for extrauterine life [1]. Postpartum care encompasses the provision of health services from the moment of placental expulsion through six weeks following delivery, with a minimum of four recommended contacts at 24 h, 48–72 h, 7–14 days, and six weeks postpartum [1]. This period is uniquely vulnerable; approximately one-third of all pregnancy-related maternal deaths occur between one week and one year postpartum, with the highest mortality concentrated in the first 42 days [2].

Globally, 260,000 women die during and following pregnancy and childbirth in 2023, with approximately 92% of these deaths occurring in low- and lower-middle-income countries [3]. Approximately 87% of global maternal deaths are concentrated in sub-Saharan Africa and southern Asia [3]. The primary causes of postpartum maternal mortality in resource-limited settings include postpartum hemorrhage, hypertensive complications, infection, and mental health disorders [4, 5]. Beyond mortality, substantial morbidity persists. Severe maternal morbidity encompasses hemorrhage, sepsis, eclampsia, and related complications that cause long-term disability and impair health-related quality of life [4]. Early detection and management of these complications during the postpartum period through quality postnatal care and accessible health services can prevent most of these deaths [3]. Postpartum mental health disorders, including postpartum depression, anxiety, and other perinatal mood and anxiety disorders, constitute a major but often unrecognized cause of postpartum morbidity in low-resource settings. In sub-Saharan Africa, the pooled prevalence of postpartum depressive symptoms ranged from 22.1% (95% confidence interval: 18.5–26.2), with regional variation from 13.5% in Tanzania to 30.6% in South Africa [6]. Beyond the mother, postpartum mental health disorders have profound consequences. Maternal depression is associated with impaired parent–infant bonding, disrupted breastfeeding, adverse neonatal feeding outcomes, and developmental delays in infants [7, 8]. The bidirectional relationship between breastfeeding and maternal mental health underscores the importance of integrated psychosocial and practical support during the early postpartum period [8].

Social support, defined as emotional, instrumental (practical), informational, and appraisal assistance provided by family, peers, and health professionals, is a critical protective factor in the postpartum period [9, 10]. Research demonstrates that decreased social support is associated with an increased risk of postpartum depression, anxiety, and impaired parent–infant bonding [10]. Conversely, women reporting adequate emotional and practical support showed improved mental health outcomes, increased breastfeeding success, and faster physical recovery [10, 11]. First-time mothers are particularly vulnerable as they lack prior parenting experience and may experience heightened uncertainty regarding infant care, feeding, and maternal well-being [12]. Studies in high-income countries have revealed that first-time mothers frequently express unmet needs for emotional support, practical assistance with household tasks, and reliable informational guidance on infant care and maternal health [12, 13].

In low- and middle-income countries, the delivery of comprehensive postpartum social support is severely constrained by health system challenges that are characteristic of fragile, conflict-affected, and vulnerable settings. Fragile and conflict-affected settings, such as Somalia, face disrupted health service organizations, limited human resources for health, inadequate infrastructure, and competing public health priorities [14, 15]. Continuity of care, a cornerstone of effective postpartum management, is frequently disrupted in these contexts because of shortages of skilled health workers, geographic barriers, insecurity, and limited facility capacity [14, 16]. Task-shifting, the deliberate delegation of health tasks from specialist to non-specialist health workers, has emerged as a necessary strategy to expand access to maternal health services; however, its implementation in postpartum care remains limited and inconsistent [17, 18]. Community health workers and trained family members can effectively deliver components of postpartum education, psychosocial support, and basic care when appropriately supervised. However, investment in their training and support systems remains minimal in many sub-Saharan African contexts [17, 18].

Somalia exemplifies the intersection of these health system challenges and profound maternal and neonatal health deficits. In Somalia, the maternal mortality ratio is 621 per 100,000 live births, among the highest globally, with recent estimates indicating that only 2.8% of women receive postnatal care, and only 32% of births are attended by skilled birth attendants [16, 19]. A recent analysis of the Somalia Health and Demographic Survey 2020 found that only 0.6% of Somali women completed the full continuum of maternity care (four or more antenatal visits, skilled birth attendance, and postnatal care within 48 h), with zero continuum coverage documented among pastoralist communities [19]. Profound inequities exist; rural and nomadic populations, less-educated women, and those with limited income or decision-making power show substantially lower maternal health service utilization [16, 19]. Postpartum complications, including hemorrhage, infection, and hypertensive disorders, remain the leading causes of maternal morbidity and mortality; however, their detection and management capacity are critically limited [16].

At Banadir Hospital, Somalia’s largest maternal and child health referral facility in Mogadishu, first-time mothers present limited prenatal preparation, variable access to skilled delivery support, and often fragmented postpartum follow-up. The sociocultural context in Somalia emphasizes traditional kinship and family-based support networks; however, the provision of specific postpartum physical support (assistance with infant care and household tasks), emotional support (reassurance, coping guidance, mental health screening), and informational support (counseling on infant feeding, danger signs, and maternal recovery) remains ad hoc and informal [16]. Health workers at Banadir Hospital reported time constraints, insufficient training in psychosocial care, and limited tools for identifying postpartum depression and other mental health concerns [16]. Community-level support is similarly fragmented: extended family members, though present, often lack knowledge of postpartum complications, infant care best practices, and mental health warning signs, limiting their capacity to provide effective support [16].

Despite the significant burden of postpartum morbidity and mortality in Somalia and the critical role of social support in preventing adverse outcomes, little is known about the specific experiences of first-time mothers in this context. Existing literature on postpartum experiences in sub-Saharan Africa, while valuable, often combines all parity groups and may not capture the distinct vulnerabilities and support needs of first-time mothers who face unique informational gaps, heightened anxiety, and greater dependence on support systems [12, 13]. No peer-reviewed qualitative studies have systematically explored how first-time mothers in Somalia experience physical, emotional, or informational support during the critical postpartum period. Such evidence is essential for (1) identifying context-specific barriers and facilitators to effective postpartum support, (2) informing health system strengthening initiatives, (3) guiding the development of culturally appropriate interventions, and (4) supporting policy initiatives to strengthen the postpartum care continuum and reduce preventable maternal and neonatal morbidity.

A phenomenological qualitative research approach is particularly appropriate for addressing this gap in evidence. Phenomenology prioritizes the lived experience and meaning-making of participants, allowing for an in-depth exploration of how first-time mothers perceive, interpret, and respond to postpartum social support within their sociocultural and health system contexts. This design enables researchers to uncover not only the presence or absence of support but also its perceived adequacy, acceptability, and impact on maternal well-being dimensions often missed by quantitative surveys. Banadir Hospital provides an ideal study site given its high volume of first-time mothers, on-site presence of skilled health professionals, and role as the primary referral facility for maternal complications in Mogadishu.

The overarching aim of this study was to explore first-time mothers’ experiences of postpartum social support at Banadir Hospital in Mogadishu, Somalia. The specific study objectives were (1) to explore first-time mothers’ experiences of postpartum physical support, including assistance with infant care, household responsibilities, and practical daily needs; (2) to explore first-time mothers’ experiences of postpartum emotional support, including reassurance, empathetic listening, mental health guidance, and emotional connection from partners, family, and health workers; and (3) to document first-time mothers’ experiences of postpartum informational support, including guidance on infant feeding, childcare, danger signs, maternal health, and health system navigation. The findings from this study will contribute crucial evidence to inform postpartum care strengthening initiatives in Somalia and similar fragile, conflict-affected settings.

## Materials and methods

### Study design

An exploratory phenomenological qualitative research design was employed to investigate first-time mothers’ lived experiences of postpartum social support at the Banadir Hospital. Phenomenology is a research approach uniquely suited to exploring and understanding how individuals perceive and interpret specific phenomena within their lived contexts [20]. This design enabled in-depth exploration of first-time mothers’ subjective experiences and perspectives regarding postpartum physical, emotional, and informational support. The phenomenological approach prioritizes the voices and experiences of participants, allowing researchers to uncover the essence of their lived experiences while capturing contextual factors that influence their perceptions. Rather than testing predetermined hypotheses, this design allowed the research team to explore systematically how first-time mothers experienced support from family members, healthcare workers, and communities during the critical early postpartum period. The exploratory nature of the study ensured that novel insights could emerge from participants’ narratives without being constrained by pre-existing theoretical frameworks, thereby providing a rich, contextualized understanding of postpartum support dynamics in the Somali health system context.

### Study setting

The Banadir Hospital, located in Mogadishu, Somalia, served as the study site. The hospital is recognized as Somalia’s largest maternal and child health referral facility, and functions as a national teaching hospital for obstetrics, gynecology, and pediatrics. The facility provides comprehensive maternal health services, including antenatal care, skilled birth attendance, postnatal care, and pediatric services, to a diverse population from both urban and rural areas of Mogadishu and surrounding regions. With an average monthly postnatal clinic attendance of approximately 175 women, the Banadir Hospital receives a high volume of first-time mothers presenting for postpartum care and immunization services. This high service volume, combined with the hospital’s role as a referral center for maternal complications, makes it an ideal setting for investigating first-time mothers’ experiences of postpartum social support. The facility’s diverse client population, representing varied socioeconomic circumstances and geographic backgrounds, provides opportunities to explore varied experiences of postpartum care and support.

### Study population

The study population comprised two distinct groups. The primary participants were first-time mothers (primiparous women) aged 18 years and above who were within six months of delivery and attending postnatal care clinics or immunization clinics at Banadir Hospital during the study period. Secondary participants were healthcare workers, including nurses, midwives, physicians, and health extension workers employed at Banadir Hospital, who provided direct maternal and neonatal care services. The inclusion criteria for first-time mothers were as follows: being in their first pregnancy and delivery, being aged 18 years or older, having a living infant, having delivered at or presented to Banadir Hospital within six months postpartum, being able to communicate in Somali, and providing written informed consent. Healthcare workers were included if they had been directly involved in providing postpartum care at Banadir Hospital for at least one year prior to the study and consented to participate. Exclusion criteria for first-time mothers included severe acute illness requiring emergency care, cognitive impairment preventing informed consent, and inability to communicate verbally in Somali. Healthcare workers with less than one year of postpartum care experience were excluded to ensure that they had sufficient experience to provide meaningful insights into maternal support provision at the facility.

### Sampling and sample size

Purposive sampling was employed to recruit study participants based on their relevant experiences and ability to provide information-rich narratives about postpartum social support. Purposive sampling ensured that participants were deliberately selected because they possessed specific characteristics and experiences that were directly relevant to the research questions. Participants were selected to represent diversity in age, educational attainment, marital status, and socioeconomic circumstances to capture their varied postpartum experiences. Data saturation, a point at which no new themes or insights emerged from additional data collection guided the determination of the final sample size. Data collection and analysis proceeded iteratively, and the research team regularly reviewed the collected data to assess whether theoretical saturation had been achieved. The study ultimately enrolled 38 participants who provided data using three distinct data collection methods. These comprised 16 first-time mothers who participated in in-depth individual interviews, 12 first-time mothers who participated in two separate focus group discussions, with six participants in each group, and 10 healthcare workers who participated in key informant interviews. 

### Data collection procedures

Data were collected using semi-structured interviews and focus group discussion guides specifically designed to explore participants’ lived experiences of postpartum physical, emotional, and informational support. The data collection tools were pretested among a similar population in Medina Hospital, Somalia. Data was collected by trained research assistants who were nurses. Semi-structured guides incorporated open-ended questions that allowed participants to describe their experiences in their own words, while focusing on the key domains of interest. All interviews and focus group discussions were conducted in Somali, the participants’ native language, to ensure optimal comprehension and allow participants to express themselves authentically and with nuances. All data collection sessions were digitally audio-recorded, with prior verbal and written consent from the participants. Field notes documenting nonverbal communication, setting observations, and researcher reflections were supplemented with audio recordings. Example questions from the in-depth interview guide included: ‘What kind of physical help did you receive at home during the postpartum period?’ “Describe the emotional support you received after giving birth,” and “What information or guidance were you given about caring for your baby and your own health?” The focus group discussion guides used parallel questions adapted for group discussion, while key informant interview checklists explored healthcare workers’ perspectives on maternal support provision, barriers to support, and recommendations for improvement. Data collection occurred over four months, from March to June 2024, allowing sufficient time for recruitment, conducting interviews and focus groups, transcription, and preliminary analysis. Each in-depth interview lasted approximately 45 to 60 min, focus group discussions lasted 60 to 90 min, and key informant interviews lasted 30 to 45 min, depending on the depth of participants’ responses and flow of discussion.

### Data management and analysis

All audio-recorded interviews and focus group discussions were transcribed verbatim into written text. Transcripts were initially prepared in Somali and then translated into English by trained bilingual translators to ensure preservation of the participant’s language while making data accessible to the broader research team. Transcripts were managed and analyzed using NVivo 11 software (QSR International, Melbourne, Australia), a qualitative data analysis platform that facilitates the systematic organization, coding, and retrieval of textual data. Data analysis employed inductive thematic content analysis, a systematic approach that involves identifying patterns and themes within data without imposing pre-existing theoretical frameworks [21, 22]. The analytical process involved several sequential steps. First, the research team familiarized themselves with the data through repeated reading of the transcripts and listening to audio recordings. Second, open coding was applied to identify meaningful units of information and assign descriptive labels to transcript segments. Third, the codes were organized into preliminary categories based on shared meanings. Fourth, the categories were refined and clustered to develop broader themes and subthemes through iterative comparison and refinement. Finally, themes were interpreted in relation to the research questions and the existing literature on postpartum social support. The iterative analytical process ensured the continuous refinement of thematic interpretations throughout the study. Through systematic coding and thematic development, three overarching themes emerged that corresponded directly to the study objectives: postpartum physical support encompassing assistance with infant care, household tasks, and maternal recovery support; postpartum emotional support encompassing reassurance, empathetic listening, mental health guidance, and emotional connection from partners, family, and healthcare providers; and postpartum informational support encompassing guidance on infant feeding, childcare practices, danger sign recognition, and health system navigation. The research team collectively reviewed all coded data and thematic interpretations to ensure accuracy, consistency, and coherence throughout the analysis.

### Trustworthiness and rigor

To ensure trustworthiness and rigor, the current study employed credibility, which ensured that the findings accurately reflect the study participants’ lived experience of postpartum social support. This was achieved through multiple strategies, including triangulation of data sources through three distinct methods: in-depth individual interviews, focus group discussions, and key informant interviews. Additionally, peer debriefing sessions were held with two experienced experts in qualitative research to promote the quality of the data, eliminate biases, and promote ethical conduct of the study. Dependability, the consistency and reproducibility of study procedures, was ensured through a comprehensive audit trail documenting all research decisions, coding frameworks, analytical choices, data management procedures, and methodological reflections. Confirmability, the degree to which findings are grounded in data rather than researcher bias or assumptions, was supported through the audit trail, reflexive journaling, triangulation of data sources, and peer debriefing. Transferability, the potential applicability of findings to other contexts, was supported through detailed, thick descriptions of the study setting, participant characteristics, data collection procedures, and contextual factors specific to Mogadishu and Somalia, enabling readers to assess the relevance of the findings to their own contexts. Additionally, adherence to the inclusion and exclusion criteria of the study helped to ensure transferability.

### Ethical considerations

This study was approved by the Clarke International University Research and Ethics Committee (Reference: CIU-REC/025/2024) prior to the commencement of recruitment or data collection activities. Written informed consent was obtained from all study participants before participation. Information sheets detailing the study objectives, procedures, potential benefits and risks, and participants’ rights were provided to all potential participants. These information sheets explicitly outlined the participants’ right to voluntary participation, the right to refuse to answer specific questions, and the right to withdraw from the study at any time without penalty or impact on their healthcare. Consent forms were reviewed by participants and signed prior to data collection. Throughout the data collection, ongoing consent was reinforced at the beginning of each session, with participants again reminded of their right to withdraw. Confidentiality and anonymity were maintained through systematic procedures. All identifiable participant information was replaced with coded identifiers (e.g., IDI 01 for the first in-depth interview participant, FGD 02 for the first focus group participant, or KII 01 for the first key informant). A master list linking participant codes to identifying information was maintained separately in a secure location accessible only to the principal investigator. All audio recordings and transcripts were stored in password-protected, encrypted computer files, and paper records containing any identifying information were kept in a locked cabinet in the research office. During data analysis, the analysis proceeded using participant codes rather than names, ensuring that no identifying information appeared in the documents. The final results and conclusions are reported using aggregated data and participant codes, with no names or identifying characteristics appearing in the final manuscript.

### Limitations of the study

This study methodology has several important limitations that warrant acknowledgment. Locating and recruiting eligible first-time mothers proved challenging because of varied postpartum clinic attendance patterns and difficulty in reaching mothers after discharge from facility-based care. This was partially mitigated by working with community health nurses employed at Banadir Hospital, who were able to assist with participant identification and recruitment, although this approach may have introduced selection bias favoring mothers with greater healthcare engagement. Some participants demonstrated an initial reluctance to discuss sensitive emotional topics, particularly experiences with mental health challenges or difficult family relationships. This limitation was addressed through careful attention to creating a safe and private interview environment, building rapport with participants through extended conversation before formal interviews, ensuring researcher neutrality and non-judgmental responses, and reassuring participants of confidentiality. The cross-sectional design of data collection during the early postpartum period limits the study to capturing experiences at a single time point rather than exploring how support experiences change over the extended postpartum year. The single-site design of recruiting exclusively from Banadir Hospital in Mogadishu may limit generalizability to other settings or populations, particularly to rural, nomadic, or internally displaced populations with different healthcare access patterns. The involvement of healthcare workers in participant recruitment may have introduced a social desirability bias, with mothers potentially providing responses they perceived as desired by healthcare providers rather than fully authentic experiences. This potential bias was mitigated through the explicit assurance of confidentiality, separation of the research team from clinical staff, and emphasis on the value of candid responses.

## Results

### Participant characteristics

A total of 38 participants, comprising 28 first-time mothers (73.7%) and 10 healthcare workers (26.3%) who served as key informants, provided contextual insights into postpartum social support at Banadir Hospital. Data were collected through in-depth interviews (IDI) with 16 first-time mothers, two focus group discussions (FGD) with 12 first-time mothers (six participants per group), and key informant interviews (KII) with 10 healthcare workers. The demographic characteristics of the participants are shown in Table 1. The majority of the participants were aged 30–39 years (52.6%), followed by those aged 20–29 years (23.7%). Nearly half (47.4%) had attained primary education, and the majority (73.7%) were married. This demographic profile reflects the broader population of postpartum mothers accessing care at Banadir Hospital, which is consistent with the maternal health service utilization patterns documented in Somalia.

**Table 1:** Demographic Characteristics of Study Participants (*N* = 38)

Variable	Category	Frequency (*n*)	Percentage (%)
Age (years)	20–29	9	23.7
	30–39	20	52.6
	40–49	6	15.8
	≥ 50	3	7.9
Education	Primary	18	47.4
	Secondary	7	18.4
	Certificate	3	7.9
	Diploma	5	13.1
	Degree	3	7.9
	Postgraduate	2	5.3
Marital Status	Single	4	10.5
	Married	28	73.7
	Separated	6	15.8

### Thematic findings

Three major themes emerged from the qualitative data analysis corresponding to the three study objectives: (1) postpartum physical support, (2) postpartum emotional support, and (3) postpartum informational support. These themes reflect first-time mothers’ lived experiences of social support during the immediate postpartum period, and highlight significant gaps in the provision, consistency, and adequacy of support. Table 2 summarizes the major themes, subthemes, and illustrative quotes.

**Table 2:** Summary of Major Themes and Subthemes

Main Theme	Subthemes Identified
Physical Support	1. Assistance with baby care; household chores 2. Spousal or family help 3. Short duration of support 4. Financial constraints
Emotional Support	1. Partner encouragement 2. Family empathy 3. Peer support groups 4. Health worker counseling 5. Mood fluctuations; inadequate support
Informational Support	1. Difficulty communicating needs 2. Newborn care guidance 3. Health complications management 4. Inadequate professional counseling

### Theme 1: Postpartum physical support

First-time mothers in this study experienced some level of postpartum physical support, primarily from family members, including mothers, mothers-in-law, and spouses. Physical support encompassed tangible assistance with infant care (carrying, bathing, and feeding), household tasks (cooking and washing clothes), and maternal recovery activities. Participants reported receiving help during the initial weeks of the postpartum period, which allowed them time to rest and recover from childbirth. One participant stated:

*“I was supported during the first months when I could hardly support myself on my own. I had my clothes washed*,* and food brought to my room.” (FGD/06)*

Another mother described the importance of family assistance in managing the demands of newborn care.

*“My baby would cry a lot in the night*,* so mother helped me carry him so that I could catch some sleep at night.” (FGD/01)*

These accounts highlight the critical role of family members in providing direct physical assistance during the early postpartum period. However, the participants reported that physical support was often short-lived and inconsistent. After the first four weeks postpartum, the level of assistance from family members declined significantly, leaving first-time mothers to manage infant care and household responsibilities independently. One mother explained:

*“The support we get is limited because my husband earns little money*,* which is not enough to pay for and hire an external helper. And perhaps my mother has her family demands*,* which also limit her support sometimes. Her support is limited to a day or two a week.” (IDI/07)*

Financial constraints have emerged as a major barrier to sustained physical support. Spouses and family members were often unable to afford external helpers, and family members faced competing work and household responsibilities, which limited their availability. Another participant highlighted the lack of family support.

*“My husband is a casual laborer who earns a small wage that he cannot afford a househelp… he also gets very exhausted with his work and cannot support with household chores.” (IDI/13)*

### Theme 2: Postpartum emotional support

Emotional support emerged as a critical but under-addressed dimension of postpartum social support among first-time mothers. Emotional support encompasses reassurance, empathetic listening, validation of maternal feelings, and encouragement from partners, family members, peers, and health care providers. Participants described emotional distress, mood fluctuations, feelings of inadequacy, and fears related to infant care and maternal recovery, consistent with the psychosocial vulnerabilities that are characteristic of the early postpartum period.

Several participants reported that emotional support was limited and primarily provided by healthcare workers rather than family members. One mother explained:

*“Whenever l lack emotional support when faced with challenging events that make me stressed*,* I find it hard to hold onto my baby or do any chores*,* my moods fluctuate*,* and this in turn affects the breastmilk flow.” (FGD/09)*

A key informant healthcare worker observed:

*“Inadequate emotional support is common among first-time teenage mothers who have inadequate knowledge and experience in childbirth. Most of the first-time mothers who present at the facility with emotional support challenges have malnourished babies.” (KII/05)*

Participants who received emotional support from their partners and extended family members reported improved coping and reduced stress. One mother shared:

*“My partner has been very helpful in ensuring that I am emotionally well by helping me babysit the baby while I execute other roles and household chores*,* or find time to sleep during the day. Whenever he is around*,* I feel less burdened.” (IDI/02)*

Another participant emphasized the value of family involvement:

*“My family members have been helpful in getting some of the would-be hard tasks done with love and care. This has helped me stay less stressed and have less negative thoughts towards the baby. Family has been the best source of relief during critical times.” (FGD/01)*

Partner involvement in postpartum support has been consistently associated with reduced maternal stress, improved mental health outcomes, and enhanced maternal-infant bonding. However, participants in this study noted that family members often lacked knowledge of postpartum mental health disorders and were unable to identify early warning signs of postpartum depression or anxiety, thus limiting their capacity to provide effective emotional support.

*“My family does not understand postpartum depression. When I was depressed*,* they thought I was being moody….They could not recognize the signs*,* they did not know how to support me emotionally.” (IDI/08)*

Peer support has emerged as an important but under-utilized source of emotional support. Participants who encountered other postpartum mothers at the health facility reported benefiting from their shared experiences and practical advice. One mother stated:

*“The existence of experienced postpartum mothers at the health facility during postpartum visits helped me a lot gain knowledge and tactics of managing the baby and the daily household chores.” (FGD/08)*

Peer support programs have been shown to reduce postpartum depressive symptoms, enhance maternal confidence, and improve breastfeeding outcomes. However, structured peer support programs were not available at Banadir Hospital, and informal peer interactions occurred sporadically, rather than systematically.

Healthcare workers have emerged as the primary formal source of emotional support for first-time mothers experiencing distress. One participant explained this as follows:

*“My main source of emotional support has been mainly through the health workers at the health facilities who provide me with counselling on how to manage stressful events and stress related to baby colic pain.” (FGD/11)*

### Theme 3: Postpartum informational support

Informational support, defined as guidance on newborn care, infant feeding, maternal health, danger signs, and health system navigation, emerged as a critical gap in postpartum care at Banadir Hospital. First-time mothers reported limited access to reliable professional health information and expressed frustration with the lack of structured counseling and education during the postpartum period.

Participants described difficulty communicating their informational needs to healthcare providers, often feeling reluctant to ask questions because of perceived hierarchical barriers within the hospital setting. One mother explained:

*“In the face of informational challenges*,* l felt laid back whenever l had questions that needed clarity. I had the impression that there were processes that one ought to follow*,* or channel inquiries through a specific hierarchical order.” (IDI/15)*

This finding reflects broader patterns of health system-related barriers to patient-provider communication, particularly in resource-constrained settings, where patient-centered care models are underdeveloped.

First-time mothers expressed strong informational needs related to newborn care, including bathing, changing diapers, cord care, feeding techniques, and recognizing infant health complications. Participants expected that such information would be provided systematically before hospital discharge, but reported receiving minimal or no guidance. One mother stated:

*“The most challenging part was doing things without prior information. l expected that information concerning childcare would all be provided at birth. At one point*,* it was clear that they were going to explain to us how to dress the baby up*,* cord care*,* how to change diapers*,* and information on dos and don’ts when handling a baby. And these were things that were important to us*,* because we didn’t know.” (IDI/06)*

Lack of newborn care information has been documented as a major contributor to maternal anxiety, delayed care seeking for infant complications, and suboptimal infant feeding practices. Maternal health literacy, the ability to access, understand, and apply health information, is strongly associated with improved neonatal outcomes, exclusive breastfeeding, and timely immunization.

The participants also expressed informational needs regarding infant health complications, particularly recognizing danger signs and knowing when to seek medical care. One mother described uncertainty and distress regarding her infant’s health.

*“When my baby was born*,* she had tremors; they [health care personnel] said you need to monitor the evolution of your newborn’s tremor. Then you are stressed because you don’t really know what you should look for what’s normal or not.” (FGD/01)*

This lack of clear guidance on identifying and responding to neonatal complications contributes to delayed care seeking and preventable morbidity and mortality. In low-resource settings, such as Somalia, where skilled health workforce shortages and limited healthcare infrastructure constrain service delivery, structured discharge counseling and postpartum follow-up systems are frequently absent.

Participants reported relying predominantly on informal sources such as family members, neighbors, and community members for health information, often receiving conflicting or inaccurate advice. Healthcare workers acknowledged the limited provision of formal informational support due to time constraints, high patient volumes, and a lack of standardized counseling protocols.

### Summary

The findings from this study revealed that first-time mothers at Banadir Hospital, Mogadishu, Somalia, experienced suboptimal postpartum social support across all three domains: physical, emotional, and informational. Physical support, while initially present, is short-lived and constrained by financial limitations and competing family responsibilities. Emotional support was limited and primarily provided by healthcare workers rather than by family members, reflecting gaps in family awareness of postpartum mental health needs. Informational support was critically insufficient, with first-time mothers reporting an unmet need for structured guidance on newborn care, maternal health, and complication recognition. These findings underscore the urgent need for health system strengthening initiatives, community-based support programs, and policy interventions to enhance the postpartum care continuum in Somalia and similar fragile conflict-affected settings.

## Discussion

This qualitative phenomenological study revealed that first-time mothers at Banadir Hospital, Mogadishu, Somalia, experience suboptimal postpartum social support across physical, emotional, and informational domains, despite the sociocultural emphasis on family-based support networks [23, 24]. While physical support, primarily from family members, was initially present, it was constrained by financial limitations and competing family responsibilities and diminished significantly after the first month postpartum [25]. Emotional support was limited and primarily provided by healthcare workers rather than by family members, reflecting family members’ insufficient knowledge of postpartum mental health needs and early warning signs [26]. Informational support was critically insufficient, with first-time mothers reporting substantial unmet needs for structured guidance on newborn care, maternal health, and recognition of complications [27].

These findings align with evidence from other sub-Saharan Africans and low-resource settings, where social support has been consistently identified as a critical determinant of postpartum well-being, mental health, and neonatal outcomes [10, 24]. A recent multi-country qualitative study across Kenya, Ghana, Zambia, Pakistan, and India documented that a lack of social support was a major barrier to women’s quality of life during the postpartum period, with economic challenges exacerbating this burden [24]. However, the findings from this study extend previous evidence by highlighting the particular vulnerabilities of first-time mothers, whose lack of prior parenting experience intensifies their dependence on reliable and consistent social support for both practical and emotional needs [11]. In the Somali context, sociocultural norms emphasizing kinship-based support systems have not translated into robust or sustained postpartum assistance, likely reflecting the intersection of poverty, ongoing humanitarian crises, and weakened community structures characteristic of fragile conflict-affected settings [16, 28].

The insufficient provision of emotional and informational support documented in this study has important implications for maternal mental health and neonatal outcomes. The bidirectional relationship between maternal emotional distress and breastfeeding impairment documented in our findings aligns with established evidence that postpartum depression and anxiety are associated with reduced breastfeeding duration, impaired infant feeding practices, and adverse neonatal growth outcomes [8, 29]. Moreover, inadequate maternal health literacy, reflected in mothers’ insufficient knowledge of newborn danger signs and care practices, contributes to delayed care seeking for neonatal complications and preventable morbidity and mortality [27, 30]. These pathways underscore why strengthening postpartum social support across all three domains represents a crucial high-impact intervention for improving maternal and neonatal health outcomes in Somalia.

Addressing the postpartum social support gaps identified in this study requires a multilevel health system and community-based interventions aligned with implementation science principles. At the health system level, capacity building of healthcare workers in psychosocial counseling, mental health screening, and patient-centered communication is essential [31, 32]. Evidence from Sub-Saharan Africa and other LMICs demonstrates that structured training using cascade models or technology-assisted approaches can enable primary and community health workers to deliver evidence-based psychosocial interventions, including depression screening, supportive counseling, and peer support facilitation [33, 34]. In Somalia, where the WHO has identified task shifting and community health worker strengthening as critical strategies for maternal health system recovery [35], formal training of healthcare workers and community health workers in postpartum psychosocial care must be integrated into pre-service and in-service training curricula. At the community level, structured peer support interventions, leveraging experienced mothers to provide informational and emotional support to first-time postpartum mothers, have demonstrated effectiveness in reducing depressive and anxiety symptoms and improving maternal well-being [34, 36]. Family engagement interventions that educate spouses and extended family members on postpartum physical needs, mental health warning signs, and infant care practices can strengthen the protective capacity of informal support networks [37]. The implementation of structured discharge counseling protocols at Banadir Hospital and other maternal referral facilities, coupled with community-based postpartum follow-up through health workers and peers, would bridge the critical information gaps identified in this study.

This study had several limitations. The qualitative exploratory design and single-site recruitment at Banadir Hospital restrict generalizability to the broader Somali population, particularly to rural and nomadic populations with limited access to the health system [38]. The relatively small sample size (*N* = 38), which is appropriate for phenomenological inquiry, limits the range of experiences captured. The cross-sectional nature of data collection during the early postpartum period (≤ 6 months) did not permit the assessment of postpartum social support trajectories or long-term outcomes for mothers and infants. Future research should employ mixed-methods designs involving larger, more geographically diverse samples of first-time mothers, longitudinal follow-up to assess the durability of postpartum support and its long-term impacts on maternal mental health and infant development, and implementation trials of culturally adapted peer support and family engagement interventions to generate rigorous evidence on the effectiveness and cost-effectiveness of context-specific support programs in Somalia and similar fragile settings [39]. This study’s strength lies in its focus on the choice of study area. Somalia has been marked by political instability, which may affect healthcare service delivery and negatively impact postpartum support. Additionally, the study employed triangulation by using different methods of data collection.

## Conclusion

This phenomenological qualitative study provides critical evidence of first-time mothers’ lived experiences of postpartum social support in Somalia, revealing significant gaps across physical, emotional, and informational domains that undermine maternal well-being and potentially compromise neonatal health outcomes. Strengthening postpartum social support in Somalia requires urgent, multi-pronged interventions, including: (1) formal healthcare workers and community health worker training in psychosocial care, mental health screening, and family centered communication; (2) implementation of structured peer support programs leveraging experienced mothers; (3) family engagement and education initiatives addressing postpartum mental health and infant care needs; and (4) structured discharge counseling and community-based postpartum follow-up systems. Such interventions, grounded in implementation science and adapted to the Somali sociocultural and health system context, are essential for advancing postpartum care quality, reducing preventable maternal and neonatal morbidity and mortality, and achieving maternal health equity in fragile, conflict-affected settings. 

## Supplementary Information

Supplementary Material 1.

## Data Availability

The datasets generated during this study are available from the corresponding author upon reasonable request and subject to ethical and privacy restrictions.
