# Supplementary material for: Lived experiences of first-time mothers receiving postpartum social support at Banadir Hospital, Mogadishu, Somalia: a phenomenological qualitative study
Source: BMC Pregnancy Childbirth. 2026 Feb 26;26:367. doi: 10.1186/s12884-026-08875-y (PMC13040759; doi:10.1186/s12884-026-08875-y)
Supplement: Supplementary file 1 — Supplementary Material 1. [file 12884_2026_8875_MOESM1_ESM.docx]

**Supplementary File**

**Data collection**

# **Focus Group Discussion Guide**

Questions

1. What were your first time experiences of postpartum social support in Bosaso, Somalia?
2. Tell us about the social support you received during pregnancy and after and a young adolescent?
3. Did you have expectations for postpartum social support during your first time birth?
4. Explain the physical support you received or experienced after birth?
5. What was your emotional support experience after birth?

- Counseling support
- Psycho-social support
- Enhanced positive feeling
- Hope provision

1. What are some of the challenges you face that hinder you from receiving social support?
2. What are the physical support challenges that you faced after giving birth/
3. What emotional support challenges did you face after giving birth?
4. What are first time mothers’ experiences of postpartum informational support in Bosaso district Somalia?

- Information on health care support
- Information on health information
- Information on child care
- Information on Hygiene
- Information on appropriate feeding
- Information on General wellness

1. What are the challenges you face as in information support in Bosaso district Somalia?

#

# **Key Informant Interview Guide for Health Workers**

Questions

1. What experiences do first-time mothers experience in terms of Physical support to you after birth?
2. What are the challenges do first-time mother’s face in physical support in Bosaso district Somalia?
3. What are the first-time mothers’ experiences of postpartum emotional support in Bosaso district Somalia in terms of
4. What are the challenges faced by first-time mothers in emotional support in Bosaso district Somalia?
5. What are first-time mothers’ experiences of postpartum informational support in Bosaso district Somalia?
6. What health system approaches have you devised to enhance the social support for first time mothers in Bosaso district Somalia?

# **In-depth Interview for Selected Mothers**

Questions

1. What experiences do you experience in terms of Physical support to you after birth?
2. What are the challenges you face in physical support in Bosaso district Somalia?
3. Are there any mechanisms to enable you to attain Physical support?, What are the mechanisms to support you physically after birth?
4. What is your experience of postpartum emotional support in Bosaso district Somalia, in terms of
5. What are the challenges your mothers face in emotional support in Bosaso district, Somalia?
6. What approaches do you devise for/ spouse/ health facility/ community in ensuring your emotional support?
7. What is your experience of postpartum informational support in Bosaso district, Somalia?
8. What are the challenges of attaining informational support on postpartum?
9. What health system/ community/ spouse approaches have been devised to enhance the social support for first-time mothers in Bosaso district Somalia?
